# Do Growing Rabbits with a High Growth Rate Require Diets with High Levels of Essential Amino Acids? A Choice-Feeding Trial

**DOI:** 10.3390/ani11030824

**Published:** 2021-03-15

**Authors:** Pablo Jesús Marín-García, Mari Carmen López-Luján, Luís Ródenas, Eugenio Martínez-Paredes, María Cambra-López, Enrique Blas, Juan José Pascual

**Affiliations:** 1 Departamento Producción y Sanidad Animal, Salud Pública y Ciencia y Tecnología de los Alimentos, Facultad de Veterinaria, Universidad Cardenal Herrera-CEU, CEU Universities, 46022 Valencia, Spain; 2 Institute of Animal Science and Technology, Universitat Politècnica de València, Camino de Vera s/n, 46022 Valencia, Spain; malolu@upvnet.upv.es (M.C.L.-L.); luiromar@dca.upv.es (L.R.); eumarpa@upv.es (E.M.-P.); macamlo@upvnet.upv.es (M.C.-L.); eblas@dca.upv.es (E.B.); jupascu@dca.upv.es (J.J.P.)

**Keywords:** rabbit, lysine, methionine, threonine, retention, digestibility

## Abstract

**Simple Summary:**

Two diets were designed to investigate the effect of the growth rate on amino acid requirements in growing rabbits: M diet (with medium levels of amino acids, following current nutritional recommendations for growing rabbits) and H diet (with high levels of lysine, sulphur amino acids and threonine compared with current nutritional recommendations). Performance, nutrient retention and digestibility (faecal and ileal) trials, as well as a choice-feeding test were conducted. We found no differences in animal performance, nutrient retention and digestibility parameters between diets, but the animals showed a preference for the M diet, expressed by a high intra-individual repeatability in preference. Our results provide useful information and nutrition tools to move towards precision protein nutrition in growing rabbits.

**Abstract:**

As a consequence of the genetic selection process in growing rabbits, there are currently fast-growing animals exhibiting an average daily gain that may exceed 45 g/d. The protein requirements of these animals, namely amino acid requirements, may differ from animals with low growth rates. The objective of this work was to evaluate growth performance, the coefficient of total tract apparent digestibility (CTTAD), the apparent ileal digestibility (AID) of amino acids and nutrient retention of fast-growing rabbits when they had access to a diet with high levels of amino acids and/or a diet formulated with current nutritional recommendations in a choice-feeding trial. To this end, two diets were formulated: the M diet following current nutritional recommendations for growing rabbits (including 8.1, 5.8 and 6.9 g/kg dry matter (DM) of total lysine, sulphur amino acids and threonine, respectively) and the H diet with higher levels of total lysine, sulphur amino acids and threonine (9.4, 6.6 and 7.8 g/kg DM, respectively). A total of 220 weaned rabbits, from a paternal line selected for the growth rate, had free access to the M diet, the H diet or both (MH) diets from 28 to 63 days of age. The CTTAD of DM, crude protein and gross energy from 49 to 53 days of age as well as the AID of amino acids of the diets at 63 days of age were determined. Protein, amino acids and energy retained in the empty body from 28 to 63 days of age were also registered. No significant differences in growth performance, CTTAD, AID and nutrient retention between dietary treatments were observed. However, animals fed the H diet showed a higher AID of cysteine (*p* < 0.05) and higher threonine retention (*p* < 0.05) compared to the M diet. As regards the choice-feeding trial, MH animals showed a higher feed intake of the M diet compared to the H diet (+8.61%; *p* < 0.001), and furthermore, more than 50% of the animals preferred the M diet throughout the experimental period (*p* < 0.05). Our results suggest that animals with high growth rates do not show significantly higher productive traits when fed the H diet compared to the M diet. As regards choice feeding, MH animals were capable of choosing their preferred diet, showing high intra-individual repeatability in preference for the M diet. It would be interesting to continue studying this behaviour of choice based on amino acid levels.

## 1. Introduction

Paternal rabbit lines selected for the growth rate are used in rabbit breeding in order to improve feed efficiency. Current breeding systems select for the average daily gain (ADG) because there is a genetic negative correlation between the ADG and feed efficiency [1,2]. As a consequence of this selection process, industrial rabbit farming involves rearing high-productive animals exhibiting an ADG that may exceed 45 g/d. According to Partridge [3], there is a positive relationship between animal weight and protein requirements (i.e., protein required for maintenance). Therefore, protein requirements of high-productive animals with a high growth rate, namely amino acid requirements, may differ from animals with a low growth rate.

Matching nutrient supply with nutrient requirements of animals is the basis of precision livestock feeding. This is necessary in the interest of safe, high-quality and efficient production, while ensuring the lowest-possible load on the environment [4]. Meeting these requirements as accurately as possible with an adequate balance in essential nutrients can furthermore impact positively animal health. The literature has shown that in growing rabbits, an excess of dietary protein can contribute to a high incidence of mucoid enteropathy [5,6,7,8]. There are other studies, however, where the relationship between dietary protein and health status in growing rabbits is unclear [9,10,11].

Current commercial nutritional recommendations for growing rabbits tend towards reducing dietary protein and increasing fibre content to avoid digestive disorders [11].In this context, high-productive animals with high growth rates are currently fed moderate levels of protein. In previous research, ref. [12,13] outlined the existence of some limiting amino acids when current moderate-protein diets are used in fast-growing rabbits.

Usually, the first limiting amino acids are lysine, sulphur amino acids and threonine for poultry, pigs and rabbits [14,15,16]. Moreover, the use of the apparent ileal digestibility (AID) of amino acids in recommendations rather than their total dietary or total tract apparent digestible content would permit a more accurate evaluation system. The use of the AID evaluation system is necessary to contribute to decrease the dietary protein content and maximize the nitrogen digestion efficiency. However, current amino acid recommendations are mainly expressed in total dietary or faecal digestible trials. Furthermore, growing rabbits have certain digestive peculiarities such as caecotrophy, and therefore its contribution to protein supply could hinder the evaluation of protein requirements itself [17,18].

Choice feeding (dietary self-selection method) is based on providing simultaneously two or more diets throughout the growing period for animals to choose from. Some choice-feeding trials carried out in broilers and pig species show that animals can choose between different diets to obtain a nutrient intake that matches their requirements [19]. These tests can therefore contribute to determining the animals’ nutrient requirements. In rabbits, choice-feeding trials have been used to investigate choices between feed and forage [20,21]. However, to the best of our knowledge, choice-feeding trials have not been performed in rabbits to address specific nutritional requirements.

The main aim of this work was to evaluate growth performance, the coefficient of total tract apparent digestibility (CTTAD) of main nutrients, the apparent ileal digestibility (AID) of amino acids and nutrient retention of fast-growing rabbits when they had access to a diet with high levels of amino acids and/or a diet formulated with current nutritional recommendations in a choice-feeding trial.

## 2. Materials and Methods

The experimental procedure was approved by the Animal Welfare Ethics Committee of the Universitat Politècnica de València (authorisation code: 2018/VSC/PEA/0116) and carried out following the recommendations of the European Group on Rabbit Nutrition [22] and Spanish Royal Decree 53/2013 on the protection of animals used for scientific purposes [23].

### 2.1. Experimental Diets

Table 1 and Table 2 show the ingredients and chemical compositions of the basal mixture and experimental diets used in this work. The two experimental diets were prepared from a common basal mixture formulated following current nutritional recommendations for growing rabbits [14], except for the first three limiting amino acids (lysine, sulphur amino acids and threonine). Both experimental diets were obtained by adding synthetic amino acids (L-lysine HCL, DL-methionine and L-threonine) to the basal mixture, resulting in an M diet with medium levels of amino acids (including 8.1, 5.8 and 6.9 g/kg dry matter (DM) of total lysine, sulphur amino acids and threonine, respectively, following current nutritional recommendations for growing rabbits) and an H diet with higher levels of lysine, sulphur amino acids and threonine (9.4, 6.6 and 7.8 g/kg DM, respectively). Amino acid dietary levels in the H diet were increased by 13–16% compared with the M diet based on [24]. A version of both diets including 5 g/kg DM of alfalfa hay marked with ytterbium was also manufactured for ileal measurements (including 0.5% of lucerne marked with ytterbium).

Experimental feeds were provided ad libitum from the start of the trial to the animals in each group in pelleted form.

### 2.2. Animals

In this study, 222 weaned rabbits (28 days of age) from the R line were used. The R line was obtained after two generations of random mating from a pool of animals of three commercial sire lines [25] and then selected by the ADG in the growing period during 38 generations. This line is characterised by a high growth rate during the growing period, and it was developed at the Institute of Animal Science and Technology of Universitat Politècnica de València (València, Spain).

### 2.3. Experimental Procedure

The experimental procedure was carried in six consecutive batches, under controlled environmental conditions (animals were kept at 15 °C to 22 °C, with a photoperiod of 12 h of light and 12 h of darkness).

At 28 days of age, 15 weaned rabbits were slaughtered by intracardiac puncture with sodium thiopental (75 mg/kg of body weight (BW)) to determine the empty body characteristics at this age. The rest of the animals (207) were housed in individual cages and assigned to one of the following experimental groups: M, with free access to the M diet; H, with free access to the H diet; and MH, with free access to both M and H diets. Mortality and morbidity (presence of diarrhoea) was controlled daily, and feed intake of each diet and the BW were registered weekly. Animals presenting any digestive anomaly, weight loss or low ingestion were discarded.

Digestibility of nutrients was only determined in groups fed M and H diets. At 42 days of age, 24 randomly selected animals (12 from M and 12 from H groups) were housed in individual metabolic cages of 52 × 44 × 32 cm^3^ and, after a 7-day acclimatisation period, a faecal digestibility trial was conducted according [26]. From 49 to 53 days of age, feed consumption was controlled and faeces produced during the whole period were collected. The faeces were stored in identified plastic bags and frozen at −20 °C until analysis. From 53 days of age, 24 randomly selected animals (12 from M and 12 from H groups) received the same diet but marked with ytterbium until slaughter at 63 days of age.

At 63 days of age, 75 randomly selected animals (25 from each experimental group) were weighed and slaughtered by intracardiac injection of sodium thiopental (75 mg/kg of BW). Slaughter was conducted between 19:00 p.m. to 23:00 p.m. to minimise the influence of caecotrophy on the composition of the digestive content.

Samples of ileal content were obtained from the distal part of the small intestine (around 20–30 cm before the ileo-caeco-colic valve) from each animal receiving the marked feeds with ytterbium (only M and H groups), frozen at −20 °C, freeze-dried and ground. The whole digestive tract was emptied and reintroduced into the body of the slaughtered animals. Empty bodies, obtained at 28 and 63 days of age (15 and 75 animals, respectively) were weighed and placed into identified plastic bags and frozen at −40 °C. Frozen empty bodies were crushed and homogenised in a cutting machine (Tecator, Abusson, France), and a sample per animal was freeze-dried and stored at −40 °C until analysis.

### 2.4. Chemical Analysis

Feed was analysed for DM, ash, crude protein (CP), ether extract (EE), neutral detergent fibre (aNDFom), acid detergent fibre (ADFom), lignin (sa), starch, gross energy (GE) and amino acid content. Faeces were analysed for DM, CP and GE, while ileal samples were analysed for DM, CP and amino acid content. Finally, empty bodies were analysed for DM, CP, GE and amino acid content. Samples were analysed according to the methods described in [27]: 934.01 for DM, 942.05 for ash, 976.06 for CP and 920.39 for EE. The aNDFom (assayed with a thermo-stable amylase and expressed exclusive of residual ash), ADFom (expressed exclusive of residual ash) and lignin (determined by solubilisation of cellulose with sulphuric acid) were analysed sequentially [28]. The GE content was determined by adiabatic bomb calorimetry (Gallenkamp Autobomb, Loughborough, UK).

The amino acid content was determined after acid hydrolysis with 6N HCL at 110 °C for 23 h, as previously described in [29], using a Waters (Milford, MA, USA) HPLC system consisting of two pumps (Mod. 515, Waters), an autosampler (Mod. 717, Waters), a fluorescence detector (Mod. 474, Waters) and a temperature control module. Aminobutyric acid was added as an internal standard after hydrolysation. The amino acids were derivatised with 6-aminoquinolyl-N-hydroxysuccinimidyl carbamate (AQC) and separated with a C-18 reverse-phase column Waters AcQ Tag (150 mm × 3.9 mm). Methionine and cystine were determined separately as methionine sulphone and cysteic acid, respectively, after performic acid oxidation followed by acid hydrolysis [30]. Ytterbium was analysed according to [27] by absorption spectrometry (Smith-Hieftje 22; Thermo Jarrell Ash, MA, USA).

### 2.5. Statistical Analysis

Data were statistically analysed using SAS software [31]. Data obtained from rabbits slaughtered at 28 days of age (empty body weight and CP, GE and amino acids contents in the empty body) were fitted to the BW at 28 days of age using linear regression equations to estimate the initial values of rabbits slaughtered at 63 days of age. Differences between the estimated values at 28 days of age and those obtained at the end of the retention trial (63 days of age) were used to estimate nutrient retention during the growing period.

Data on animal performance (BW, ADG, daily feed intake (DFI) and feed conversion ratio (FCR)) were analysed using the MIXED model of SAS, taking into account the lack of homoscedasticity of data. The model included the diet, batch, week and their interactions as fixed effects. Data from the CTTAD and the AID and nutrients retained in the empty body of the animals (energy, protein and amino acids) were analysed using the GLM model of SAS, including the diet as the only fixed effect. With the aim to determine the preference for a certain diet of each individual animal during the whole experimental period, a *t*-test analysis was performed. In this way, the intra-individual preference was defined.

## 3. Results

The effect of the experimental diets on the CTTAD of the main nutrients and the AID of DM, CP and amino acids is presented in Table 3. No significant difference in the CTTAD and AID was observed between M and H diets, with the exception of the AID of cysteine, which was significantly higher in the H compared with the M diet (+39%).

Regarding the health status of the rabbits during the experimental period, there were no significant differences in mortality and morbidity between the different experimental groups (M, H and MH), mortality being, on average, 23% and morbidity 11%, due to an outbreak of epizootic rabbit enteropathy (data not shown).

Table 4 shows the growth performance data of healthy animals. In general, there were no significant differences in the BW, DFI, ADG and FCR between the three experimental groups. However, the global FCR of the MH group was higher compared with the H group (+5%; *p* = 0.0077).

Energy, protein and amino acids retained in the empty body during the growing period for each experimental group are presented in Table 5. There were no significant differences in retained nutrients between groups, except for threonine, which was higher in the H group compared with the M (+12%; *p* = 0.0345) and MH (+11%; *p* = 0.0236) groups.

**Figure 1:**
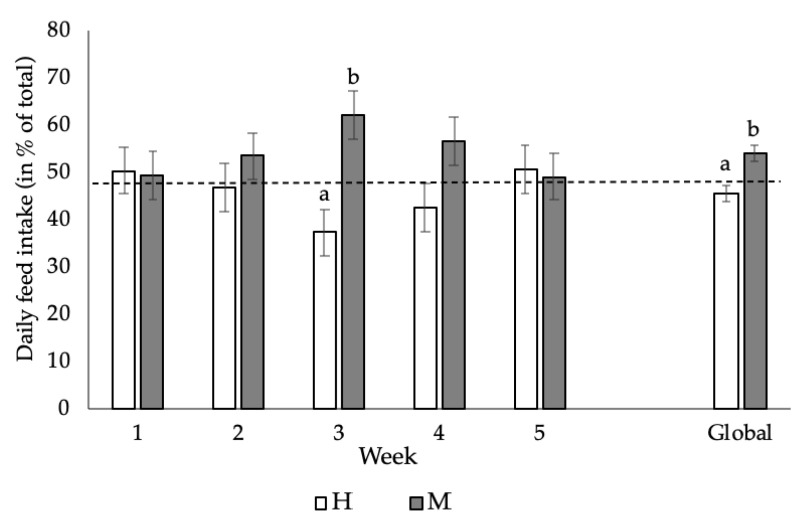
Daily feed intake (mean and standard error) of growing rabbits fed the M diet (following current nutritional recommendations for growing rabbits, including 8.1, 5.8 and 6.9 g/kg dry matter (DM) of total lysine, sulphur amino acids and threonine, respectively) and the H diet (with higher levels of total lysine, sulphur amino acids and threonine: 9.4, 6.6 and 7.8 g/kg DM, respectively) during the five-week choice-feeding trial, as well as during the whole growing period (global). ^a,b^ Means within different letters were significantly different (*p* < 0.05). Week 1 (28–35 days), week 2 (35–42 days), week 3 (42–49 days), week 4 (49–56 days) and week 5 (56–63 days).

Figure 1 shows the preference in the weekly feed intake of the animals subjected to the choice-feeding trial (MH diet). Globally (from 28 to 63 days of age), animals showed a preference for the M diet, resulting in a higher DFI of the M diet compared with the H diet (+8.61%; *p* = 0.00134). This difference, however, was not significant during the weekly periods, except for the third week (+25%; *p* = 0.0011). Regarding the intra-individual effect, more than 50% (*p* < 0.03) of the animals showed a clear and repeated preference for the M diet throughout the experimental period.

## 4. Discussion

Our hypothesis was that rabbits with high growth rates would have higher growth needs and that therefore the H diet would show better performance results and higher preference in the choice-feeding trial. To discuss our initial hypothesis, we will discuss the dietary effect and the choice-feeding results separately.

### 4.1. Differences between Diets (M and H)

The CTTAD and AID values obtained in this study agree with those of other trials carried out with this same paternal genetic line and using similar diets [13]. Additional supplementation of the first three limiting amino acids did not affect the CTTAD of the main nutrients. These results agree with others previously obtained, where lysine, methionine or threonine supplementation did not affect the digestibility of DM, CP, and GE [32,33,34]. However, recent studies have observed that dietary supplementation with methionine from 2.4 to 4.9 g/kg [35] or from 5.6 to 8.3 g/kg [36] could increase the CTTAD of DM, CP and GE in growing rabbits. In any case, ref. [37] already indicated that supplementing with synthetic amino acids does not necessarily improve the apparent digestibility of nutrients.

Although the values obtained for the AID of lysine, methionine and threonine were slightly higher for the H diet compared with the M diet (+6.1, +5.0 and +6.1 percentage points), only cysteine showed a significantly higher value (+16.3 percentage points; *p* = 0.0247). This result may be a consequence of the higher digestibility of the synthetic amino acids (lysine, methionine and threonine), which were added to the H diet to a greater extent [33,38,39]. In broilers, ref. [40] also observed that by increasing the content of sulphur amino acids from 85% to 100% above requirements (using dietary supplementation with synthetic DL-methionine), the birds showed an increased content of cysteine in plasma, amongst other amino acids. Cysteine intestinal transport involves two carrier-mediated processes [41] that are selectively inhibited by other amino acids with small polar side chains (such as threonine) or large non-polar side chains (such as methionine). Therefore, an increase in the ileal digestibility of cysteine does not seem to be related to an increase in its transport. The improvement in cysteine digestion and the higher AID values observed for the rest of the amino acids in the H diet should be related to greater pancreatic activity. Usually, an increase in the intake of a nutrient (such as protein or amino acids) induces an increase in the secretion of the enzymes that hydrolyse it (such as trypsin, chymotrypsin and elastase, amongst others; [42]).

On average, the animals showed a high ADG (54 g/d) and an adequate FCR (2.8). These data agree with reported growth performance data for the same paternal genetic line [43,44,45,46]. As regards the main research question posed in this work, i.e., whether growing rabbits with high growth rates require diets with high levels of essential amino acids, in our study, growing rabbits fed the H diet did not show a significant improvement in their BW, ADG, DFI and FCR compared with those fed the M diet. These results indicates that additional supply of lysine, sulphur amino acids and threonine does not provide an advantage with respect to the current amino acid recommendations for fattening rabbits [14,45,46] developed a tool, based on the plasmatic urea nitrogen (PUN) content, to determine possible dietary amino acid unbalances in rabbit feed. Using the same diets as in this work, Marín-García et al. [24] observed that PUN levels were similar for both M and H diets. Similar values of PUN levels could be an indicator of a comparable fitting to the animals’ requirements and a similar available amount of protein and energy for growth [47,48]. However, ref. [24] observed that PUN levels were minimised and growth performance maximised with a diet containing the same level of lysine as the M diet, the same methionine level as the H diet but lower threonine levels (5.7 g/kg DM). Therefore, an inadequate supply of amino acids, both due to a lack or excess, leads to an increase in the catabolism of amino acids and PUN levels. Under these conditions, a higher contribution of all amino acids would not lead per se to an improvement in the productive indices, and it is recommended to seek the adequate contribution of each one of them.

As expected, similar growth performance data also lead to similar energy, protein and amino acid retention in the empty body [13,49,50]. In any case, there was a trend towards a higher amount of protein (+0.7 g/d; *p* = 0.09), and most of the amino acids were retained in the empty bodies in animals fed the H diet, although statistically significant only in the case of threonine (+12%; *p* = 0.04). These results could explain why animals fed the H diet also presented the best FCR values, and could point out the need for specific amino acid recommendations for fast-growing rabbits.

### 4.2. Choice Feeding

Logically, since there were no differences in production performance data between diets, when animals were allowed to feed on both M and H diets simultaneously (MH diet), we found similar productive traits. In fact, the only difference observed (a reduced FCR with the MH diet compared with the H diet) could be related to the improvement in the FCR obtained with the H diet in the first week after weaning (*p* = 0.05). Marín-García [51], already observed that the use of feed that is more adjusted to the requirements of fast-growing animals improves growth performance, which would explain the improvement in the FCR observed with the H diet.

Results from the choice-feeding trial indicated that when offered both diets simultaneously, the animals made a choice and preferred the M diet (Figure 1) [52], in a choice-feeding trial with broilers, using diets either 10% above or below the amino acid recommendations, also showed that the birds preferred the diet with a lower amino acid content. Our results have shown for the first time that growing rabbits can discriminate between diets based on differences in amino acid concentrations [21], conducted a choice-feeding trial in which growing rabbits were fed a diet poor in methionine and lysine, and the animals had two options: to drink water enriched or not enriched with these deficient amino acids. Their study concluded that animals had a clear preference for the solution enriched with the deficient amino acids.

Our results indicated that preference for the M diet starts in the second week of the growing period, being highly marked in the third and fourth weeks. In our opinion, preference of the growing rabbits for the M diet over the H diet may be explained by two facts. Firstly, young rabbits may consume a higher amount of the H diet post-weaning because their protein and amino acid requirements are higher at that age; however, as they grow, they increase the consumption of the M diet as their protein requirements decrease [53] Secondly, the incidence of epizootic rabbit enteropathy could have played a relevant role. The symptoms of this disease started in the second week after weaning, with the third and fourth weeks being the ones with the highest incidence. Under such circumstances, the animals most affected by the outbreak may have preferred the M diet. If this were the case, our results would indicate the ability of growing rabbits to decide the type of diet based on not only their nutritional but also health needs.

Finally, this study shows that the M diet seems to be more nutritionally balanced than the H diet. The global predilection for the M diet, although existing, is not as marked as the individual preference for it. Weekly data showed that more than 50% of the animals preferred the M diet throughout the experimental period. This is the first time this feeding behaviour has been described. This preference could not be attributable to differences in the growth rate (as described in broilers) [54].

## 5. Conclusions

In the light of the results presented in this study and under the conditions described herein, we can conclude that a joint dietary increase over the current nutritional recommendations of the first three limiting amino acids (lysine, sulphur amino acids and threonine) does not improve growth performance in growing rabbits selected for high growth rates. Therefore, each amino acid must be separately fitted to the adequate proportion in order to obtain further improvements in the growth performance of fast-growing animals. It would be interesting to delve into the choice of diets for their nutritional value (specifically amino acids) for future research. However, although growing rabbits showed a similar intake of both M and H diets in the choice-feeding trial, MH animals were capable of choosing their preferred diet, showing high intra-individual repeatability in the preference for the M diet.

## Figures and Tables

**Table 1:** Ingredients (g/kg) of the basal mixture.

Ingredients	Basal Mixture
Wheat bran	300
Straw	175
Sunflower meal	167
Alfalfa hay	148
Beet pulp	120
Barley	42
Sugar beet molasses	29
Palm oil	4.5
Sodium chloride	4.8
Calcium carbonate	2.6
L-lysine HCL	2.4
Monocalcium phosphate	0.2
Vitamin/Trace element mixture ^1^	5.2

^1^ Contains per kg of feed: vitamin A, 8375 IU; vitamin D3, 750 IU; vitamin E, 20 mg; vitamin K3, 1 mg; vitamin B1, 1 mg; vitamin B2, 2 mg; vitamin B6, 1 mg; nicotinic acid, 20 mg; choline chloride, 250 mg; magnesium, 290 mg; manganese, 20 mg; zinc, 60 mg; iodine, 1.25 mg; iron, 26 mg; copper, 10 mg; cobalt, 0.7 mg; butyl hydroxylanysole and ethoxiquin mixture, 4 mg.

**Table 2:** Chemical composition (g/kg DM) of the basal mixture and experimental diets.

	Basal Mixture	M	H
Dry matter (DM; g/kg)	896	905	902
Ashes	73.2	72.5	72.7
Crude protein	157	155	156
Ether extract	26.7	26.4	26.5
Neutral detergent fibre	473	468	470
Acid detergent fibre	275	272	273
Acid detergent lignin	58.1	57.5	57.7
Gross energy (MJ/kg DM)	-	18.50	18.64
Digestible energy ^1^ (DE; MJ/kg DM)	-	11.21	11.35
Digestible protein ^1^ (DP; g/kg DM)	-	117	118
DP/DE (g DP/MJ DE)	-	10.44	10.39
**Amino acids**			
Alanine	5.81	5.75	5.77
Arginine	8.25	8.17	8.20
Aspartic acid	12.7	12.6	12.6
Cysteine	2.36	2.37	2.37
Glutamic acid	26.7	26.4	26.5
Glycine	7.43	7.36	7.38
Histidine	3.02	2.99	3.00
Isoleucine	5.42	5.37	5.38
Leucine	9.59	9.49	9.53
Lysine	7.29	8.10 ^2^	9.40 ^2^
Methionine	2.64	3.43 ^2^	4.23 ^2^
Phenylalanine	6.07	6.01	6.03
Proline	8.44	8.36	8.38
Serine	6.63	6.56	6.59
Sulphur amino acids	5.00	5.80	6.60
Threonine	5.04	6.90 ^2^	7.80 ^2^
Tyrosine	2.99	2.96	2.97
Valine	7.42	7.35	7.37

M diet: following current nutritional recommendations for growing rabbits (with 8.1, 5.8 and 6.9 g/kg dry matter (DM) of total lysine, sulphur amino acids and threonine, respectively); H diet: with higher levels of total lysine, sulphur amino acids and threonine (9.4, 6.6 and 7.8 g/kg DM, respectively). ^1^ Experimentally determined according to Pérez et al. (1995). Using 14 healthy growing rabbits of 49 days of live per diet weaned at 28 days. Faeces were collected during 4 days after a period of 7 days of adaptation to diets. ^2^ Values were obtained by adding L-lysine HCL, DL-methionine and L-threonine, respectively.

**Table 3:** Coefficient of total tract apparent digestibility (CTTAD) of the main nutrients (n = 24) and apparent ileal digestibility (AID) of dry matter, crude protein and amino acids (n = 32) from animals fed the experimental diets (M and H) during the fattening period (average ± standard deviation).

	M		H		*p*-Value
**CTTAD**					
Dry matter	0.547	±0.005	0.549	±0.005	0.8194
Crude protein	0.751	±0.007	0.755	±0.008	0.6854
Gross energy	0.606	±0.005	0.609	±0.005	0.6831
**AID**					
Dry matter	0.338	±0.035	0.368	±0.037	0.6425
Crude protein	0.576	±0.027	0.602	±0.027	0.5199
Alanine	0.592	±0.031	0.605	±0.036	0.7760
Arginine	0.805	±0.019	0.810	±0.021	0.8565
Aspartic acid	0.618	±0.031	0.655	±0.035	0.4338
Cysteine	0.416	±0.047	0.579	±0.050	0.0247
Glutamic acid	0.781	±0.021	0.800	±0.023	0.6488
Glycine	0.345	±0.047	0.365	±0.052	0.7643
Histidine	0.753	±0.025	0.748	±0.028	0.8862
Isoleucine	0.658	±0.032	0.690	±0.036	0.5004
Leucine	0.664	±0.032	0.697	±0.036	0.4997
Lysine	0.708	±0.029	0.769	±0.032	0.1717
Methionine	0.741	±0.023	0.795	±0.025	0.1210
Phenylalanine	0.691	±0.029	0.717	±0.033	0.5646
Proline	0.678	±0.021	0.693	±0.023	0.6492
Serine	0.521	±0.034	0.540	±0.038	0.7148
Threonine	0.645	±0.025	0.706	±0.028	0.1217
Tyrosine	0.549	±0.033	0.573	±0.037	0.6389
Valine	0.622	±0.031	0.657	±0.034	0.4431

M diet: following current nutritional recommendations for growing rabbits (with 8.1, 5.8 and 6.9 g/kg dry matter (DM) of total lysine, sulphur amino acids and threonine, respectively); H diet: with higher levels of total lysine, sulphur amino acids and threonine (9.4, 6.6 and 7.8 g/kg DM, respectively).

**Table 4:** Performance traits of animals fed the experimental diets (M, H and MH) during the fattening period (n = 140) (average ± standard deviation).

	M	H	MH	*p*-Value
**Body weight (g)**				
Day 28	607 ± 27.7	581 ± 21.4	610 ± 28.9	0.5886
Day 35	922 ± 27.7	904 ± 23.5	917 ± 28.9	0.8689
Day 42	1323 ± 27.7	1318 ± 27.2	1326 ± 28.9	0.9732
Day 49	1758 ± 27.7	1737 ± 30.0	1750 ± 28.9	0.8569
Day 56	2155 ± 27.7	2135 ± 32.3	2130 ± 28.9	0.7949
Day 63	2534 ± 27.7	2503 ± 36.5	2486 ± 28.9	0.4453
**Daily feed intake (g/d)**				
Week 1	79 ± 2.30	76 ± 2.43	78 ± 3.24	0.6154
Week 2	121 ± 3.30	124 ± 2.78	124 ± 2.66	0.7596
Week 3	165 ± 2.90	157 ± 3.27	164 ± 3.72	0.1036
Week 4	194 ± 3.50	190 ± 3.54	194 ± 3.39	0.6358
Week 5	214 ± 4.58	205 ± 5.10	208 ± 4.37	0.4020
Global	155 ± 2.45	150 ± 2.53	153 ± 2.56	0.4070
**Average daily growth (g/d)**				
Week 1	44.18 ± 1.24	45.63 ± 0.95	43.36 ± 1.53	0.3670
Week 2	56.55 ± 1.55	58.42 ± 1.41	58.06 ± 1.21	0.6314
Week 3	62.48 ± 1.24	60.42 ± 1.48	60.06 ± 1.02	0.2691
Week 4	55.84 ± 1.41	56.34 ± 1.42	53.84 ± 1.64	0.4768
Week 5	53.42 ± 1.34	52.36 ± 1.54	50.35 ± 1.82	0.3866
Global	54.49 ± 0.75	54.63 ± 0.72	53.14 ± 0.80	0.2697
**Feed conversion ratio**				
Week 1	1.76 ± 0.04	1.65 ± 0.04	1.80 ± 0.07	0.0504
Week 2	2.14 ± 0.03	2.12 ± 0.03	2.13 ± 0.04	0.8480
Week 3	2.65 ± 0.05	2.61 ± 0.04	2.73 ± 0.05	0.1833
Week 4	3.49 ± 0.07	3.38 ± 0.06	3.58 ± 0.08	0.1149
Week 5	4.00 ± 0.08	3.94 ± 0.08	4.08 ± 0.11	0.5673
Global	2.81 ± 0.03 ^ab^	2.73 ± 0.03 ^a^	2.87 ± 0.04 ^b^	0.0242

M diet: following current nutritional recommendations for growing rabbits (with 8.1, 5.8 and 6.9 g/kg dry matter (DM) of total lysine, sulphur amino acids and threonine, respectively); H diet: with higher levels of total lysine, sulphur amino acids and threonine (9.4, 6.6 and 7.8 g/kg DM, respectively); MH diet: includes both M and H diets. Means within a row with different letters were significantly different at *p* < 0.05. Week 1 (28–35 days), week 2 (35–42 days), week 3 (42–49 days), week 4 (49–56 days) and week 5 (56–63 days).

**Table 5:** Energy (MJ/d), protein (g/d) and amino acids (g/d) retained in the empty bodies of animals fed the experimental diets (M, H and MH) (n = 75) during the fattening period (average ± standard deviation).

	M		H		MH		*p*-Value
Energy (MJ/d)	0.414	±0.016	0.443	±0.016	0.403	±0.015	0.1581
Protein	9.797	±0.288	10.489	±0.298	9.680	±0.285	0.0927
Alanine	0.471	±0.016	0.497	±0.016	0.460	±0.016	0.2402
Arginine	0.691	±0.027	0.739	±0.028	0.669	±0.027	0.1680
Aspartic acid	0.701	±0.031	0.742	±0.032	0.680	±0.030	0.3470
Cysteine	0.177	±0.010	0.183	±0.011	0.183	±0.010	0.8704
Glutamic acid	1.116	±0.046	1.227	±0.048	1.110	±0.046	0.1252
Glycine	0.807	±0.028	0.807	±0.029	0.758	±0.028	0.3192
Histidine	0.194	±0.011	0.196	±0.012	0.175	±0.011	0.3117
Isoleucine	0.321	±0.011	0.349	±0.012	0.325	±0.011	0.1476
Leucine	0.642	±0.022	0.698	±0.023	0.643	±0.022	0.1179
Lysine	0.561	±0.022	0.619	±0.023	0.565	±0.022	0.1143
Methionine	0.191	±0.009	0.195	±0.010	0.202	±0.009	0.6557
Phenylalanine	0.318	±0.011	0.336	±0.012	0.324	±0.011	0.4935
Proline	0.463	±0.019	0.475	±0.019	0.456	±0.018	0.7456
Serine	0.391	±0.018	0.417	±0.019	0.382	±0.018	0.3495
Threonine	0.344	±0.014 ^a^	0.384	±0.014 ^b^	0.341	±0.014 ^a^	0.0439
Tyrosine	0.264	±0.011	0.280	±0.011	0.280	±0.011	0.4609
Valine	0.435	±0.015	0.471	±0.015	0.442	±0.015	0.1747

M diet: following current nutritional recommendations for growing rabbits (with 8.1, 5.8 and 6.9 g/kg dry matter (DM) of total lysine, sulphur amino acids and threonine, respectively); H diet: with higher levels of total lysine, sulphur amino acids and threonine (9.4, 6.6 and 7.8 g/kg DM, respectively); MH diet: includes both M and H diets. ^a,b^ Means within a row with different letters were significantly different at *p* < 0.05.

## Data Availability

Data is contained within the article.
